# A novel growing rod technique to treat early-onset scoliosis (EOS): a step-by-step 2D surgical video

**DOI:** 10.3171/2020.1.FocusVid.19683

**Published:** 2020-01-01

**Authors:** Rodrigo Navarro-Ramirez, Oded Rabau, Alisson Teles, Susan Ge, Abdulaziz Bin Shebreen, Neil Saran, Jean Ouellet

**Affiliations:** Spine Surgery and Scoliosis Group, McGill University Health Centre, Montreal, Quebec, Canada

**Keywords:** early-onset scoliosis, EOS, growing rods, growth restriction, modified Luqué technique, video

## Abstract

Early-onset scoliosis (EOS) correction techniques have evolved slowly over the past 40 years and still remain a challenge for the spine surgeon. Avoiding spinal fusion in these patients is key to decreasing morbidity and mortality in this population.

Current treatments for EOS include both conservative and surgical options. The authors present the modified Luqué technique that has been performed at their institution for the past decade. This modified technique relies on Luqué’s principle, but with newer “gliding” implants through a less disruptive approach. The goal of this technique is to delay fusion as long as possible, with the intent to prevent deformity progression while preserving maximal growth.

Normally, these patients will have definitive fusion surgery once they have reached skeletal maturity or as close as possible. Out of 23 patients until present (close to 4-year follow-up), the authors have not performed any revision due to implant failure. Three patients have undergone final fusion as the curve progressed (one patient, 4 years out, had final fusion at age 12 years; two other patients had final fusion at 3 years). These implants, which have the CE mark in Europe, are available in Canada via a special access process with Health Canada. The implants have not yet been submitted to the FDA, as they are waiting on clinical data out of Europe and Canada.

In the following video the authors describe the modified Luqué technique step-by-step.

The video can be found here: https://youtu.be/k0AuFa9lYXY.

## Transcript

Today we will be presenting to you a surgical technique to implement a self-growing rod construct termed “modern Luqué trolley.”

Case presentation consists of a neuromuscular deformity that has been progressing over time, essentially collapsing spine, gradual getting worse from the age of 3 to the age of 5; essentially has a collapsing spine. Patient deformity is relatively flexible on traction film.

Typical setup; we place the patient in halo and femoral traction.

Retractors are inserted just above the metaphyseal flare. K-wires are placed across the shaft of the femur.

The patient is then position prone on proper bolsters. Preoperatively we proceed to mark the pedicle screws, giving us the layout of the surgical plan. This is comprised of typically two to three levels of solid anchors proximally and distally using standard fixed pedicle screws. Fluoroscopy guidance is utilized to identify the pedicle screws on the surface of the skin. Bed is typically put in reverse Trendelenburg, allowing us additional retraction.

Skin incision is centered over the spinal processes thus allowing that no implant is found directly underneath the incision. In proximal and distal anchors, it is important to proceed with subperiosteally dissection as we plan to fuse the proximal and distal anchors. The interspinous ligament, your proximal anchor point, must be spared, allowing us to minimize the risk of junctional kyphosis.

Distally subperiosteally dissection; more importantly here, as we are trying to spare the joints, one must not resect the capsule off it. We go and dissect out to the transverse processes. By doing so, again we are minimizing the risk of spontaneous fusion.

The exposure consists of a transmuscular approach. We detach superficial fascia along the spinal processes. This is achieved by placing a Kelly just off the midline, detaching the superficial fascia. This is all the way up, allowing us to develop the right plane, equivalent to Wiltse approach, which we leave a cuff of muscle on to the spinal processes. The fascia is reflected and we identify the transverse processes. Once the transverse processes are identified, entry point is found and pedicle screw can be inserted.

At this point we tend to use a trolley, as these will be the gliding anchors. Assembly of the screw into the screwdriver is fairly straightforward. Under fluoroscopic guidance and freehand technique, we are able to insert the pedicle screws. Position is confirmed, confirming appropriate levels. We tend to use fluoroscopic x-rays, as our proximal and distal anchors are crucial and a lot of strain will be placed across these screws.

In the illustrated case we use monoaxial screws here, again allowing to cantilever the correction to the maximum. Here we are showing osteotomy of the inferior facet giving us nice decortication bilaterally, again allowing us a solid fusion mass. Inferior facet is burred, using a 3-mm burr; pedicle entry point is inserted, and freehand awl is used; pedicle screw track is created; pedicle screws are then inserted.

Satisfied with our proximal and distal solid fixations, the bony area exposed is then decorticated for fusion. We then proceed to dissect the paraspinal musculature. The dissection is taken with great care, leaving a nice cuff of bone onto the laminas. The only the site that is exposed is really across the transverse processes.

In this case, the patient had a severe osteopenia. Hence, in order to protect her proximal screws, we decided to augment the fixation using a sublaminar wire at the most proximal fixation. Such fixation allows for better prevention of screw pullout in the axial fashion.

We then turned our attention to bending the rods. Conceptually, rods should be bent with a matching contour across the overlapping segment. Great care should be taken to optimize this matching as this eases both rod insertion but also further axial growth, as illustrated, both in the coronal and sagittal plane. The idea is that the rods will be gliding (over the gliding screws), maintaining an appropriate sagittal profile.

Here we are illustrating how both rods are inserted: correct technique is that we highly recommend to capture the rods across the gliding anchors, using the dual rod pusher. One is able to see quite well rods into the gliding saddles of the trolley gliding vehicle screws.

It is critical that the cable tie is bound down; once both rods have been well seated into the saddle of the gliding anchors, this is achieved by combination of rod rotation, a bit of manual correction of the deformity, facilitating having both rods parallel. Once the apex has been captured by the trolley gliding vehicle’s screws, we then turn our attention to the actual correction maneuver for the deformity. The apex is cantilevered across midline with a rod de-rotation and then reduction to the anchors proximally and distally. This should be achieved in a gradual fashion to minimize the risk of screws’ pullout, as illustrated. One can see that both rods are de-rotated, the spine is corrected. Once the rod has been placed in the appropriate coronal/sagittal plan, then the proximal and distal rods are fixed to the fixed angle screws proximally and distally. Here we are using AO USS system mechanism, allowing us to get low-profile caps.

Similar rod contouring is done; similar reduction in the concavity is also done. Once completed the gliding cable ties are tensioned and cut.

We then proceed to decorticate the bone proximally and distally across our fixed anchors.

Closer allows us to mobilize the paraspinal musculature.

Final intraop full spine x-ray is taken confirming appropriate correction and well balance of the spine. The wound is irrigated using 3 L of saline, proximal and distal anchors are bone grafted, and then the paraspinal musculature is reapproximated in running fashion. We use a self-locking suture, allowing us to reapproximate with less tension.

Pre- and postop x-rays are revealing a good correction and well-balanced spine.

### Time Points

0:34 Preoperative x-ray

0:46 Intraoperative setting

0:56 Positioning

1:12 Intraoperative skin marking

1:37 Initial skin incision

1:55 Surgical exposure

2:40 Transmuscular approach

2:55 Preparing entry point of cephalad pedicle screws

3:02 Assemble trolley

3:10 Pedicle screw insertion

3:40 Apex region—preparing entry point and insertion of monoaxial pedicle screws

3:52 Osteotomy of inferior facets

4:12 Preparing entry point of caudal pedicle screws

4:40 Dissection of the fascia

4:50 Augmentation with sublaminar wire

5:12 Demonstrating the gliding technique

5:24 Insertion of rods

6:02 Rod rotation

6:28 Deformity correction

6:55 Decortication

7:32 Postcorrection and final x-rays

7:41 Placement bone graft

7:45 Wound closer
